# Analysis of reassortant and intragenic recombination in Cypovirus

**DOI:** 10.1186/s12985-020-01321-1

**Published:** 2020-04-06

**Authors:** Zhendong Zhang, Ning Li, Chengxiang Hou, Kun Gao, Xudong Tang, Xijie Guo

**Affiliations:** 1 School of Biotechnology, Jiangsu University of Science and Technology, Zhenjiang, 212018 Jiangsu China; 2 Sericultural Research Institute, Chinese Academy of Agricultural Sciences, Zhenjiang, 212018 Jiangsu China; 3 College of Animal Science and Veterinary Medicine, Shan Dong Agricultural University, Taian, China

**Keywords:** Cypovirus, Reassortment, Recombination

## Abstract

Cypoviruses (CPVs) are RNA viruses with segmented double-stranded genome and major pathogens of various insects, including economic insects like silkworms and pest insects for agricultural crops and forests. Genome reassortment and recombination are common phenomenon for viruses as a mechanism to expand host range and increase virulence. In the present study, we analyzed the reassortant and recombination events for CPVs. The results showed that two genome segments (S1 and S4) of BmCPV1-YN shared higher nucleotide identity with the corresponding segment of BmCPV1-I while others were all more closely to BmCPV1-SZ, suggesting BmCPV1-YN was originated from reassortant events between BmCPV1-I and BmCPV1-SZ. Recombination analyses revealed that S6 of BmCPV1-YN was a recombinant segment derived from BmCPV1-I and BmCPV1-SZ, and S10 of DpCPV1 was a recombinant segment emerged from BmCPV1-I and LdCPV1. Our findings provide the evidence for the fact that CPVs could undergo reassortant and recombinant events and enrich the knowledge about etiology and molecular epidemiology of CPVs.

## Main text

Cypoviruses (CPVs) are important viral pathogens that infect the midgut epithelial cells of insects, cause larval developmental retardation and decreased fecundity, and have been isolated from more than 250 insect species of Lepidoptera, Hymenoptera, Diptera and Coleoptera [1, 2]. CPVs are segmented double-stranded RNA (dsRNA) viruses, belonging to the genus *Cypovirus* in the subfamily *Spinareoviridae* and family *Reoviridae*, which have so far been classified into 22 elecropherotypes based on their electrophoretic migration patterns [3, 4]. There are usually 10 dsRNA segments in the genome, but some CPVs consist of 9, 11, 12 and even 16 segments [4–6]. For each segment, a high identity was revealed among viruses classified in the same electropherotype, however, no nucleotide sequence identity was found among CPVs of distinct electropherotypes [5]. Reassortant and recombination have been confirmed in various RNA viruses and several DNA viruses as a mechanism to adapt to changing environment, expand host range and increase virulence, resulting in the genetic diversity of viruses [7–9]. Reassortant and intragenic recombination events have also been reported in most viruses of *Reoviridae*, such as bluetongue virus (BTV), African horse sickness virus (AHSV), rotaviruses (RVs) and so on [10–12]. It is essential for two or more viruses to infect the same host cells, so that their genetic material might be exchanged for reassortment. Interestingly, it has been confirmed that insects could be co-infected with several CPVs [13], but there was so far no analysis or evidence of reassortant and recombination for CPVs. In the present study, we first analyzed the majority of genomic sequences of CPVs deposited in National Center for Biotechnology Information (NCBI) and then identified their reassortant and intragenic recombination events.

About 200 sequences of different segment of CPVs were acquired from GenBank database, and 15 CPV isolates have been determined for their complete genome sequences (Table S1). To better understand the relationship of CPV strains, firstly, we conducted the sequence comparative analysis by using ClustalW in Lasergene software (DNASTAR Inc., Madison, USA). Higher nucleotide identity was found among CPV strains of the same electropherotype and isolated from the same host species, for example, each genomic segment of CPV1s isolated from silkworm, *Bombyx mori* (i.e. BmCPV1-I, BmCPV1-SZ and BmCPV1-YN) share 88.6–99.8% nucleotide identity with each other. The lower nucleotide identity was found among CPVs isolated from different host species (each segment of BmCPV1-I and DpCPV1 share 78.7–90.1% nucleotide identity) although they are classified in the same electropherotype. No or few nucleotide sequence identities was found among CPVs of different electropherotypes just as described before [5]. Besides, the protein encoded by the same segment may be different among different CPV electropherotypes, for example, S1 of BmCPV1 encodes major capsid protein whereas S1 of HaCPV14 encodes RNA-dependent RNA polymerase (RdRP). Therefore, it is more meaningful to analyze the reassortant and recombination events among CPV isolates within the same electropherotype.

**Fig. 1:**
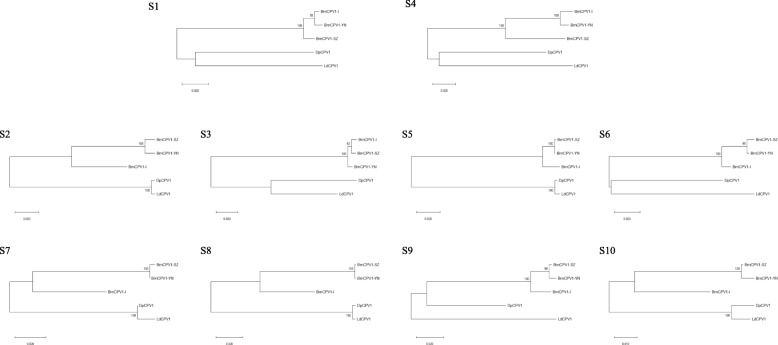
Phylogenetic analyses of CPV1. The phylogenetic trees were constructed based on the nucleotide sequences of each segment of CPV1. Reliability of the tree was assessed by bootstrap analysis of 1000 replications. Scale bar indicates nucleotide substitutions per site

**Fig. 2:**
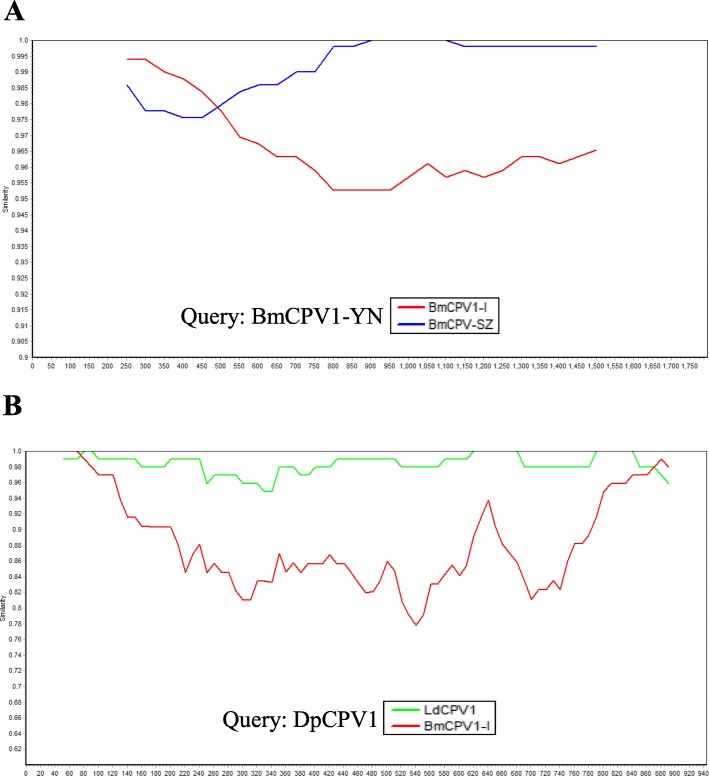
Intragenic recombination analysis. A S6 of BmCPV1-YN; B S10 of DpCPV1. Comparisons of genetic similarity between recombinant segment and parental segment were made using SimPlot. The S6 of BmCPV1-YN (**a**) and S10 of DpCPV1 (**b**) were chosen as query sequence

Each genomic segment of five strains in CPV1, three strains in CPV5 and three strains in CPV14 were compared with each other to analyze the reassortant events. Based on the results of nucleotide identity by using ClustalW in Lasergene software (DNASTAR Inc., Madison, USA), the genomic sequences of 8 segments (S2, S3, S5-S10) in BmCPV1-YN share the highest identity (98.3–99.8%) with the corresponding segments of BmCPV1-SZ, whereas S1 and S4 of BmCPV1-YN show the highest identity (98.3, 98.2%) with BmCPV1-I (Table 1), suggesting BmCPV1-YN might undergo reassortant event between BmCPV1-I and BmCPV1-SZ. To further confirm the result, the phylogenetic tree was constructed based on each segment of CPV1 genomes by the MEGA 6 software using the maximum likelihood method with a General Time Reversible (GTR) model of nucleotide substitution and bootstrap tests of 1000 replicates [14]. As shown in Fig. 1, BmCPV1-I, BmCPV1-SZ and BmCPV1-YN are clustered into a larger branch, 8 segments (S2, S3, S5-S10) of BmCPV1-YN are closely related to BmCPV1-SZ, while S1 and S4 of BmCPV1-YN are closer to BmCPV1-I. These findings clearly indicate that BmCPV1-YN is a reassortant virus emerged from segment exchange between BmCPV1-I and BmCPV1-SZ, with 8 genome segments (S2, S3, S5-S10) from BmCPV1-SZ and 2 segments (S1 and S4) from BmCPV1-I. Complete genome sequences of three strains in electropherotype 5, HaCPV5-C, OpCPV5 and TpCPV5, were also analyzed. Each segment of HaCPV5-C genome exhibits higher identity (82.9–99.1%) with OpCPV5, except for S10. The sequence of S10 of HaCPV5-C shows highest identity (99.8%) with EsCPV5 (GenBank accession no. J04338), but the relationship between these two viruses is still unclear due to the unavailability of other segment sequence of EsCPV5. Compared to the high nucleotide identity between HaCPV5-C and OpCPV5, TpCPV5 shows lower identity (40–80%) with either HaCPV5-C or OpCPV5, and TpCPV5 is clustered into a separate branch based on the phylogenetic tree (data not shown), which indicates that no reassortant events among these three strains. According to the similar analysis, no reassortment is identified among HaCPV14, LdCPV14 and TaCPV14. The intragenic recombination of individual segment within the same electropherotype was further analyzed by using RDP v4.16 and confirmed by using the software SimPlot v3.5.1 [15] and the blast results in NCBI. Two recombinant segments, S6 of BmCPV1-YN and S10 of DpCPV1, were identified (Fig. 2). S6 of BmCPV1-YN is a recombinant segment derived from the recombination event between BmCPV1-I and BmCPV1-SZ, and S10 of DpCPV1 was emerged from the recombination event between BmCPV1-I and LdCPV1.

**Table 1:** Nucleotide identities (%) between BmCPV1-YN and BmCPV1-I, BmCPV1-SZ

Strains	Segments									
	S1	S2	S3	S4	S5	S6	S7	S8	S9	S10
BmCPV1-I	98.3	89.4	98.7	98.2	97.5	97.2	88.9	89.7	96.7	92.1
BmCPV1-SZ	97.8	98.3	98.8	90.2	99.8	99.3	99.7	99.8	99.2	99.4

**Fig. 3:**
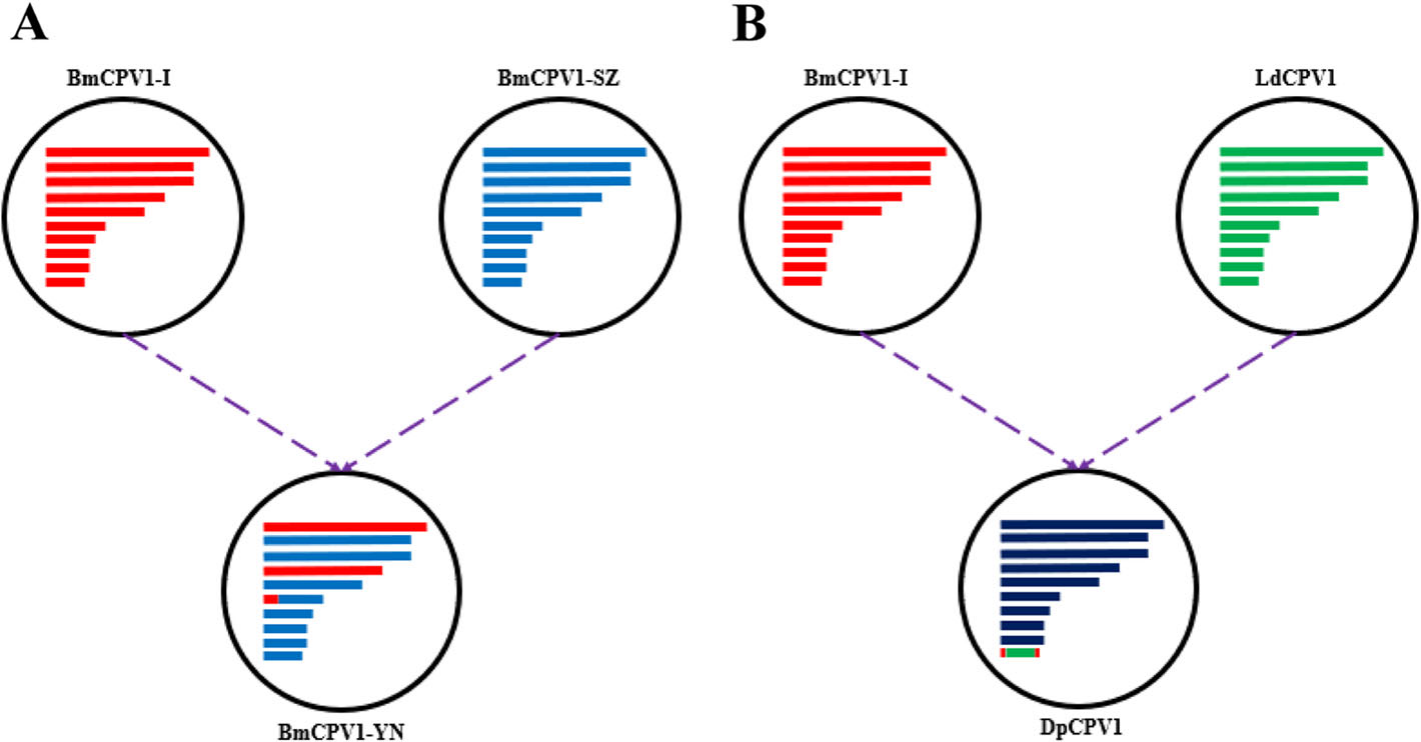
Reassortant and intragenic recombination model. **a** BmCPV1-YN; **b** DpCPV1. The individual segment is shown as “rectangle” with different colors and the length of “rectangle” indicates the size of segments

Reassortant and recombination are very important mechanism for virus evolution to escape host immune surveillance, adapt to changing environment, expand host range and so on, which has been analyzed and reported in most viruses of *Reoviridae*. The recombination events of BTV was documented in 2010 [10] and Ngoveni gave the evidence of intragenic recombination in AHSV in 2019 [12]. A study by Chen firstly reported on the occurrence of reassortant and recombinant virus between human and pig RVA strains [16]. As dsRNA viruses belonging to *Reoviridae*, some CPVs can infect several kinds of insects, while some insects can also be infected by different CPVs. Therefore, the hypothesis of reassortant and recombination events among CPVs was proposed. To our best knowledge, nucleotide similarity among CPVs of different electropherotypes is lower, therefore only the reassortant and recombination events in CPVs within the same electropherotype were analyzed in the present study. Our results show that BmCPV1-YN is a reassortant virus between BmCPV1-I and BmCPV1-SZ, and segment 6 of BmCPV1-YN is produced by recombination events between BmCPV1-I and BmCPV1-SZ (Fig. 3a). So far, only three CPV strains were isolated from the silkworm, *Bombyx mori*. As in other viruses reported in the similar papers, more reassortant and intragenic recombination events might be also possible in BmCPVs if more virus strains are identified and more sequences are deposited to NCBI in the future. It is also possible that BmCPV1-YN might be a resultant virus derived from reassortant events of some other intermediate cypoviruses, but we could not compare BmCPVs with CPVs isolated from other insect because it is not clear whether those CPVs with available genome sequence are infectious to *Bombyx mori*. The evidence of interspecies reassortant and recombination events of rotavirus have been reported [16]. Here the segment 10 of DpCPV1 is identified as a recombinant sequence derived from BmCPV1-I and LdCPV1 (Fig. 3b). It has been reported that carboxy terminus of BmCPV polyhedron is very important for its nuclear localization and modification of crystallization pattern [17], so the recombination events within the S10 terminus of DpCPV1 may be also associated with the function of polyhedron. Although more than 200 sequences were analyzed in the present study, the rate of reassortant and recombination events among CPVs are relatively low, which may be due to few available CPVs sequences within same electropherotype, lower degree of genetic compatibility or the poor competitiveness of nascent virus. Besides, for influenza A viruses and rotaviruses, the reassortant has contributed to viral pathogenic and zoonotic potentials, so the pathogenicity of reassortant and recombinant CPVs and ongoing epidemiological surveillance are required to be explored in the future.

## Supplementary information

**Additional file 1: Table S1.** GenBank accession number of each segment of 15 CPV strains. The GenBank accession number of each segment of 15 CPV strains were acquired from GenBank database. Note: BmCPV1-I, *Bombyx mori* CPV 1 strain I, Japan; BmCPV1-SZ, *Bombyx mori* CPV 1 isolate Suzhou, China; BmCPV1-YN, *Bombyx mori* CPV 1 isolate Yunnan, China; DpCPV1, Dendrolimus punctatus CPV 1; LdCPV1, *Lymantria dispar* CPV 1; IiCPV2, Inachis io CPV 2; HaCPV5-C, Heliothis armigera CPV 5 isolate China; OpCPV5, Orgyia pseudotsugata CPV 5; TpCPV5, Thaumetopoea pityocampa CPV 5; HaCPV14, Heliothis armigera CPV 14; LdCPV14, *Lymantria dispar* CPV 14; TaCPV14, Thyrinteina arnobia CPV 14; TnCPV15, *Trichoplusia ni* CPV 15 (segment 11: NC_002566); DpCPV22, Dendrolimus punctatus CPV 22 (segment 11–16: KJ191114, KJ191115, KJ191116, KJ191117, KJ191118, KJ191119); DnCPV-NC, Daphnis nerii CPV isolate Nanchang, China.

## Data Availability

All sequences obtained in this study are available in GenBank.

## Abbreviations

AHSV: African horse sickness virus
Bm: *Bombyx mori*
BTV: bluetongue virus
CPV: cypovirus
Dp: *Dendrolimus punctatus*
dsRNA: double-stranded RNA
Es: *Euxoa scandens*
Ha: *Heliothis armigera*
Ld: *Lymantria dispar*
NCBI: National Center for Biotechnology Information
Op: *Orgyia pseudotsugata*
RDP: Recombination Detection Program
RdRP: RNA-dependent RNA polymerase
RVs: rotaviruses
Ta: *Thyrinteina arnobia*
Tp: *Thaumetopoea pityocampa*
