# Supplementary material for: Analysis of reassortant and intragenic recombination in Cypovirus
Source: Virol J. 2020 Apr 6;17:48. doi: 10.1186/s12985-020-01321-1 (PMC7132967; doi:10.1186/s12985-020-01321-1)
Supplement: Supplementary file 1 — Additional file 1: Table S1. GenBank accession number of each segment of 15 CPV strains. The GenBank accession number of each segment of 15 CPV strains were acquired from GenBank database. Note: BmCPV1-I, Bombyx mori CPV 1 strain I, Japan; BmCPV1-SZ, Bombyx mori CPV 1 isolate Suzhou, China; BmCPV1-YN, Bombyx mori CPV 1 isolate Yunnan, China; DpCPV1, Dendrolimus punctatus CPV 1; LdCPV1, Lymantria dispar CPV 1; IiCPV2, Inachis io CPV 2; HaCPV5-C, Heliothis armigera CPV 5 isolate China; OpCPV5, Orgyia pseudotsugata CPV 5; TpCPV5, Thaumetopoea pityocampa CPV 5; HaCPV14, Heliothis armigera CPV 14; LdCPV14, Lymantria dispar CPV 14; TaCPV14, Thyrinteina arnobia CPV 14; TnCPV15, Trichoplusia ni CPV 15 (segment 11: NC_002566); DpCPV22, Dendrolimus punctatus CPV 22 (segment 11–16: KJ191114, KJ191115, KJ191116, KJ191117, KJ191118, KJ191119); DnCPV-NC, Daphnis nerii CPV isolate Nanchang, China. [file 12985_2020_1321_MOESM1_ESM.docx]

**Table S1. GenBank accession number of each segment of 15 CPV strains.**

| Segments  Strains | S1 | S2 | S3 | S4 | S5 | S6 | S7 | S8 | S9 | S10 |
| --- | --- | --- | --- | --- | --- | --- | --- | --- | --- | --- |
| BmCPV1-I | AF323781 | AF323782 | AF323783 | AF323784 | AB035732 | AB030014 | AB030015 | AB016436 | AF061199 | M19112 |
| BmCPV1-SZ | GU323605 | GQ924586 | GQ924587 | GU323606 | GQ294468 | GQ294469 | GQ150538 | GQ150539 | GQ924588 | GQ924589 |
| BmCPV1-YN | KR704197 | KR704195 | KR704198 | KR704199 | KR704200 | KR704201 | KR815454 | KR704202 | KR704203 | KR704196 |
| DpCPV1 | AY163247 | AY147187 | AY167578 | AF542082 | AY163248 | AY163249 | AY211091 | AY211092 | AY211093 | AY211094 |
| LdCPV1 | NC_003016 | NC_003017 | NC_003018 | NC_003019 | NC_003020 | NC_003021 | NC_003022 | NC_003023 | NC_003024 | NC_003025 |
| IiCPV2 | NC_023491 | NC_023486 | NC_023492 | NC_023487 | NC_023488 | NC_023493 | NC_023489 | NC_023494 | NC_023490 | NC_023495 |
| HaCPV5-C | NC_010670 | NC_010669 | NC_010668 | NC_010667 | NC_010666 | NC_010665 | NC_010662 | NC_010664 | NC_010663 | NC_010661 |
| OpCPV5 | KC588356 | KC588357 | KC588358 | KC588359 | KC588360 | KC588361 | KC588362 | KC588363 | KC588364 | KC588365 |
| TpCPV5 | KP217033 | KP217034 | KP217035 | KP217036 | KP217037 | KP217038 | KP217039 | KP217040 | KP217041 | KP217042 |
| HaCPV14 | DQ242048 | DQ388474 | DQ388475 | DQ388476 | DQ388477 | DQ017080 | DQ010326 | DQ010325 | DQ010324 | DQ010323 |
| LdCPV14 | AF389452 | AF389453 | AF389454 | AF389455 | AF389456 | AF389457 | AF389458 | AF389459 | AF389460 | AF389461 |
| TaCPV14 | MF161423 | MF161424 | MF161425 | MF161426 | MF161432 | MF161427 | MF161428 | MF161429 | MF161430 | MF161431 |
| TnCPV15 | NC_002557 | NC_002558 | NC_002559 | NC_002567 | NC_002560 | NC_002561 | NC_002562 | NC_002563 | NC_002564 | NC_002565 |
| DpCPV22 | KJ191104 | KJ191105 | KJ191106 | KJ191107 | KJ191108 | KJ191109 | KJ191110 | KJ191111 | KJ191112 | KJ191113 |
| DnCPV-NC | NC_040440 | KX509952 | NC_040447 | NC_040444 | NC_040445 | NC_040443 | NC_040446 | NC_040442 | NC_040441 | KX509951 |
